# Dr. Laura Marcu on Serendipity, Science, and the Power of Interdisciplinary Vision

**DOI:** 10.1117/1.BIOS.3.1.010501

**Published:** 2026-03-02

**Authors:** Travis Sawyer

**Affiliations:** a University of Arizona, College of Optical Sciences, Tucson, Arizona, United States

## Abstract

Professor Laura Marcu (UC Davis Departments of Neurological Surgery and Biomedical Engineering), Director the NIH-NIBIB P41 National Center for Interventional Biophotonics Technologies (NCIBT), discusses her career in biophotonics in an interview with Biophotonics Discovery Editor Travis Sawyer.

**Figure:**
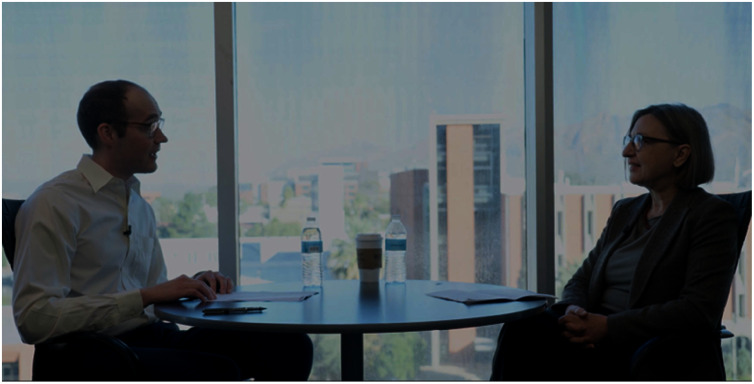
*(Right) Professor Laura Marcu (UC Davis Departments of Neurological Surgery and Biomedical Engineering), Director the NIH-NIBIB P41 National Center for Interventional Biophotonics Technologies (NCIBT), discusses her career in biophotonics with* Biophotonics Discovery *Editor Travis Sawyer (left). View a video recording of the interview at https://doi.org/10.1117/1.BIOS.3.1.010501*

When Dr. Laura Marcu reflects on her path into biophotonics, she doesn’t describe a straight line. Instead, she paints a picture of curiosity, serendipity, and the courage to follow unexpected opportunities, traits that have shaped her into one of the field’s most influential voices.

Born and raised in Romania, Marcu grew up in a world where mathematics and physics were not just academic subjects but gateways to understanding how things worked. She recalls building her own flashlight as a child—an early hint of the engineer she would become. Her high school’s shift toward a math-and-physics-heavy curriculum during the communist era nudged her further toward technical disciplines, eventually leading her to study mechanical engineering with a specialization in fine and precision mechanics at the Polytechnic University of Bucharest. It was there that she first encountered optics and optical instrumentation design.

Her journey took a dramatic turn in the early 1990s when she moved to the United States after the fall of Romania’s communist regime. California, known to her only through movies, became home as she pursued a PhD in biomedical engineering at the University of Southern California. The transition was eye-opening: she found American engineering education more applied, more connected to real-world problems, and rich with interdisciplinary collaboration.

A pivotal moment came when her PhD advisor introduced her to the laser center at Cedars-Sinai Medical Center in Los Angeles. In the mid-1990s, the center was a hub of early biomedical optics innovation including developments in fluorescence spectroscopy, Raman spectroscopy, photodynamic therapy, and experimental endoscopic systems. Working alongside surgeons, engineers, and scientists, Marcu discovered the interface that would define her career: the space where engineering meets medicine.

That environment also launched her unexpectedly into academia. After completing her PhD, she had no plans to pursue a faculty career. But her postdoctoral advisor, surgeon-scientist Warren Grundfest, encouraged her to write an NIH R01 proposal based on her dissertation work in fluorescence lifetime spectroscopy for atherosclerosis. She assumed it was unrealistic. Instead, it scored in the top percentile—an early success that propelled her into leading her own research group.

Mentorship, she emphasizes, has been central to her trajectory. Beyond Grundfest, she highlights the influence of Martin Gundersen, a USC professor known for his work in quantum electronics and plasma physics. Gundersen, she says, taught her the value of thinking beyond disciplinary boundaries, surrounding oneself with people who challenge assumptions, and aiming higher than seems possible. His encouragement helped her embrace the idea of an academic career she had never envisioned for herself.

Today, Marcu leads a thriving research program at UC Davis and directs the National Center for Interventional Biophotonics Technologies. Her work in fluorescence lifetime imaging (FLIM) has advanced dramatically over the past decade, driven by both scientific insight and rapid improvements in instrumentation. What began as a dissertation topic has expanded into a suite of clinical and preclinical applications, from cardiovascular diagnostics to surgical guidance.

But her impact extends beyond the lab. Leadership roles, such as co-leading the biomedical technology program within the UC Davis Cancer Center and contributing to SPIE initiatives, have given her a broader view of how to build programs, unite researchers across departments, and create lasting institutional structures. She describes leadership as learning to “connect the dots” not only in science but among people, resources, and shared goals.

Outside of research, Marcu is far from one-dimensional. She loves travel, classical music, tennis, skiing, and, perhaps most unexpectedly, fencing. She took up the sport after moving to Davis, drawn to its blend of strategy, quick thinking, and focus.

Her advice to early-career researchers is both practical and philosophical: learn to step back and see the bigger picture; develop your own vision; build cohesive teams across disciplines; and cultivate the ability to shift between detail and strategy. In a field as interdisciplinary as biophotonics, she argues, success depends on aligning diverse expertise toward a shared purpose.

Dr. Laura Marcu’s story is one of curiosity meeting opportunity, of technical rigor meeting clinical need, and of a scientist who continues to illuminate new paths—both literally and figuratively—for the next generation of biophotonics innovators.

## Supplementary Material

10.1117/1.BIOS.3.1.010501.s01

## Supplementary Material

A transcript of the interview that provided the basis of this summary is available in the Supplementary Material. The full interview is also available in podcast form at *Biophotonics Discovery: The Podcast*, available wherever you get your podcasts.

