# Supplementary material for: Dr. Laura Marcu on Serendipity, Science, and the Power of Interdisciplinary Vision
Source: Biophotonics Discov. 2026 Mar 2;3(1):010501. doi: 10.1117/1.BIOS.3.1.010501 (PMC13227150; doi:10.1117/1.BIOS.3.1.010501)
Supplement: Supplementary file 1 [file BIOS_003_010501_SD001.pdf]

Biophotonics Discovery Interview with Dr. Laura Marcu
Conducted by Travis Sawyer on 4 December 2025

Travis Sawyer:
Hello, everyone. My name is Travis Sawyer. I'm an assistant professor of Optical Sciences at the University of Arizona, and an associate editor for *Biophotonics Discovery*, a new journal published by SPIE.

We are conducting a series of discussions and interviews with leaders in our field to learn more about their careers, gain insight from their wisdom, and understand what they see as some of the most exciting research topics in the near future.

It is my great pleasure to welcome Dr. Laura Marcu. Dr. Marcu is a professor of Biomedical Engineering at the University of California, Davis, and Director of the National Center for Interventional Biophotonics Technologies. Laura, thank you for being here today.

Laura Marcu:
Thank you very much for the invitation.

Travis Sawyer:
To begin, could you tell us a little about your background, where you grew up, and how your career began?

Laura Marcu:
I was born in Romania and completed my early education at the Polytechnic Institute in Bucharest. My initial training was in engineering, which later became closely connected to my work in biophotonics.

I moved to the United States in the early 1990s, shortly after the collapse of the communist regime. I completed my PhD in Biomedical Engineering at the University of Southern California.

Travis Sawyer:
What was that transition like, moving from Europe to the United States at that time?

Laura Marcu:
It was a very interesting and eye-opening experience, both culturally and academically. I moved directly to California, which I had mostly known from movies. I very much enjoyed the applied approach to education here, especially the emphasis on connecting mathematics and physics to real-world applications.

Travis Sawyer:
How did you first become interested in biophotonics?

Laura Marcu:
My path into biophotonics was somewhat serendipitous. I have always been drawn to

physics and mathematics. As a child, I enjoyed experimenting with flashlights and even built my own using a battery, wires, and a bulb—initially to read after bedtime without my parents noticing.

In high school, the curriculum shifted toward math and physics, which further strengthened my interests. I initially considered medical school, but organic chemistry was not a good fit for me. Instead, I chose mechanical engineering, where I was exposed to optics, precision mechanics, and optical instrumentation.

After graduation, I worked in a research institute designing spectrometers. When I moved to the United States, I joined the Biomedical Engineering program at USC. My advisor collaborated with the laser center at Cedars-Sinai Medical Center, where I was first exposed to what we now call biophotonics—then referred to as biomedical optics.

That environment, which brought engineers, scientists, and clinicians together daily, strongly shaped my thinking and career.

Travis Sawyer:
You now lead a very productive research program. How did your research directions evolve early in your career?

Laura Marcu:
When I finished my PhD in the late 1990s, I did not initially plan to pursue an academic career. I accepted a postdoctoral position at Cedars-Sinai Medical Center to continue my thesis work.

Shortly after joining, I was encouraged to submit an NIH R01 grant based on my PhD research on fluorescence lifetime spectroscopy applied to atherosclerosis. Against all expectations, the proposal received a very high score. Receiving that grant early in my career forced me to quickly grow into an independent researcher and shaped the evolution of my research program.

Travis Sawyer:
You've mentioned the importance of mentors. Could you share an example?

Laura Marcu:
One particularly influential mentor was Martin Gunderson, a professor emeritus at USC. Although his background was in quantum electronics and plasma physics, he encouraged unconventional thinking and helped me see science in a broader historical and philosophical context.

He taught me the importance of thinking beyond traditional boundaries, surrounding oneself with people who challenge you, and aiming higher than you think is possible. He also encouraged me to pursue an academic career at a time when I had not planned to do so.

Travis Sawyer:
As a mentor yourself, what advice do you give early-career researchers?

Laura Marcu:
It is important to learn how to zoom out and see the bigger picture, while also being able to zoom in on details. Developing a clear vision and learning how to connect ideas is essential.

Equally important is learning how to build and manage a team, especially in interdisciplinary fields like biophotonics. Success depends on creating a cohesive group where people from different backgrounds work toward shared goals.

Travis Sawyer:
Could you talk about your current research, particularly in fluorescence lifetime imaging and clinical translation?

Laura Marcu:
My work began with fluorescence lifetime spectroscopy during my PhD and expanded over time into imaging and multimodal systems. Advances in lasers, detectors, and instrumentation enabled us to translate these techniques into clinical environments.

We have integrated fluorescence lifetime imaging with other modalities such as ultrasound, photoacoustics, and optical coherence tomography to provide complementary information.

Travis Sawyer:
You are currently working on image-guided brain tumor surgery. Where are you in the translation process?

Laura Marcu:
We are now conducting systematic clinical studies in patients. We are able to acquire fluorescence lifetime imaging data in near real time and integrate it with preoperative MRI, which is conventionally used in neurosurgical procedures. This integration allows for more precise tumor margin identification.

We apply this technology during open craniotomies and also through biopsy needles equipped with fiber optics, enabling better targeting of diagnostic samples.

Travis Sawyer:
Are you also continuing work in cardiovascular applications?

Laura Marcu:
Yes. Cardiovascular imaging was the focus of my PhD work and remains an important research area. Implementing fluorescence lifetime techniques in coronary arteries is challenging due to catheter size, blood flow, and speed requirements. After extensive benchtop and animal studies, we are now pursuing FDA approval to begin patient studies.

Travis Sawyer:
What advice do you have for students and early-career researchers interested in biophotonics?

Laura Marcu:
First, build a strong scientific foundation and let your work speak for itself. Leadership opportunities will follow naturally.

Engage with the community, attend conferences, volunteer, review papers, and seek mentors who are already in leadership roles. View service as a meaningful contribution rather than an obligation.

Travis Sawyer:
Looking ahead, what excites you most about the future of biophotonics?

Laura Marcu:
Biophotonics has reached a level of maturity where computational methods, artificial intelligence, and integrated photonics can play transformative roles. AI can assist not only in data analysis but also in instrument design. Integrated photonics will enable more compact and deployable systems, particularly in challenging clinical environments.

Travis Sawyer:
What challenges do you see for the field in the next decade?

Laura Marcu:
Sustained funding and resources are always a concern. Equally important is training the next generation of scientists and engineers who can bridge physics, engineering, biology, and clinical implementation. Developing this workforce is both a challenge and an opportunity.

Travis Sawyer:
Thank you so much for your time and insights, Laura. And thank you to everyone watching. Stay tuned for future interviews in this series with other leaders in biophotonics.
